# Small Molecule Membrane Transporters in the Mammalian Podocyte: A Pathogenic and Therapeutic Target

**DOI:** 10.3390/ijms151121366

**Published:** 2014-11-18

**Authors:** Cristina Zennaro, Mary Artero, Vittorio Di Maso, Michele Carraro

**Affiliations:** 1 Department of Medical, Surgery and Health Sciences, University of Trieste, Trieste 34149, Italy; E-Mail: czennaro@units.it; 2 Nephrology and Dialysis Unit, Cattinara Hospital, Trieste 34149, Italy; E-Mails: mary.artero@aots.sanita.fvg.it (M.A.); vidimaso@gmail.com (V.D.M.)

**Keywords:** transporters, podocyte, focal segmental glomerular sclerosis, penicillin G, P-glycoprotein, dexamethasone, cyclosporine, thiazolidinediones

## Abstract

The intriguingly complex glomerular podocyte has been a recent object of intense study. Researchers have sought to understand its role in the pathogenesis of common proteinuric diseases such as minimal change disease and focal segmental glomerular sclerosis. In particular, considerable effort has been directed towards the anatomic and functional barrier to macromolecular filtration provided by the secondary foot processes, but little attention has been paid to the potential of podocytes to handle plasma proteins beyond the specialization of the slit diaphragm. Renal membrane transporters in the proximal tubule have been extensively studied for decades, particularly in relation to drug metabolism and elimination. Recently, uptake and efflux transporters for small organic molecules have also been found in the glomerular podocyte, and we and others have found that these transporters can engage not only common pharmaceuticals but also injurious endogenous and exogenous agents. We have also found that the activity of podocyte transporters can be manipulated to inhibit pathogen uptake and efflux. It is conceivable that podocyte transporters may play a role in disease pathogenesis and may be a target for future drug development.

## 1. Introduction

As a lipid bilayer, the cell membrane is intrinsically impermeable to polar molecules [1]. This property promotes structural and functional integrity of the living cell, since random diffusion of the cytoplasmic contents, most of which are polar in nature, is prevented. However, the cell must obtain certain polar nutrients from the environment, such as glucose and amino acids, and thus specific transport mechanisms have evolved to “carry” these molecules across membranes. In particular, active transport mechanisms, those which carry a substance in the direction of an increasing concentration gradient, serve several vital functions: extracting nutrients for energy generation, regulation and maintenance of metabolic steady states by adjusting to fluctuations in the external environment, maintaining constant cell volume and osmotic pressure and so forth. Mediated transport across membranes is analogous to enzyme catalysis [2]—such systems are substrate-specific, demonstrate saturability with the substrate, may be inhibited competitively or noncompetitively, and are genetically determined. In addition, active membrane transport—as opposed to passive transportߞis vectorial, in the sense that the substrate is either transported into (uptake) or out (efflux) of the cell, but the same transporter does not do both [1]. 

**Figure 1:**
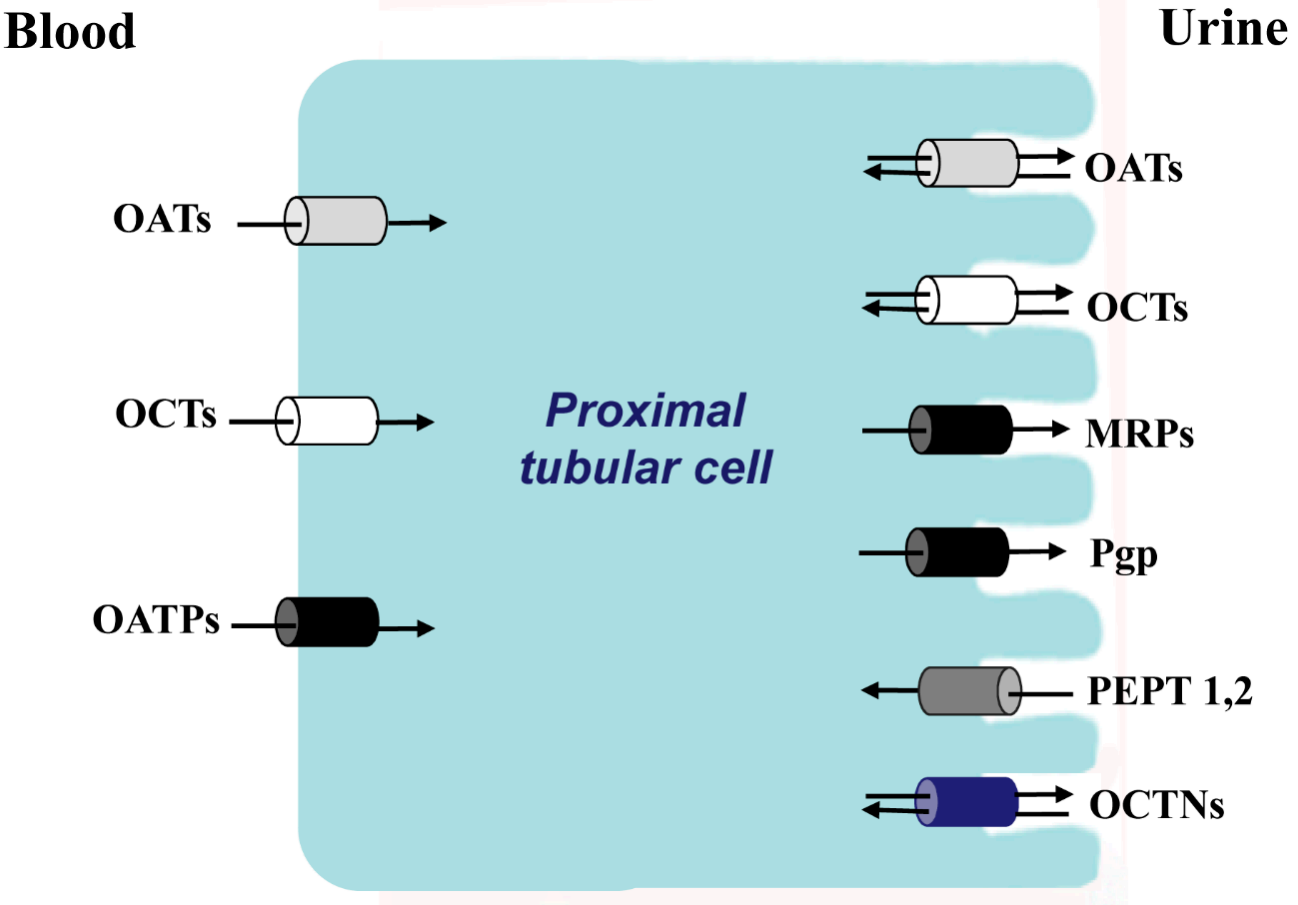
The principal families of membrane transporters in proximal tubular cells. In white color, organic cations transporters; in gray color, organic anionic transporters; in black color, anions transporters that also transport cations, zwitterions and neutral substances; in dark gray color, oligopeptides transporters; in blue zwitterion transporters.

With the discovery in the 1950s of sodium- and potassium-stimulated ATPase as a driving force for coupled membrane transport [3], many different animal tissues particularly rich in ATPase activity have been studied, including excitable cells (brain, nerve, muscle), intestinal epithelium, liver, salivary glands, and renal proximal tubular cells (see below). Besides its role in electrolyte and fluid homeostasis, the proximal tubule is recognized as the principal organ for elimination of endogenous and exogenous organic anions, cations and neutral compounds [4] which would prove toxic if allowed to accumulate (Figure 1). Thus, active transport in the proximal tubule is a vital property—accounting for approximately 60–80 per cent of total renal energy usage [1]—which has been the focus of traditional pharmacological research in the kidney 

Our interest in membrane transport of the glomerular visceral epithelial cell—also known as the podocyte—was stimulated by the recent identification of small molecule transporters that may contribute to the protein barrier function of the glomerulus apart from the well known filtration mechanisms of the slit diaphragm-basement membrane complex. As such, podocyte transporters may be involved in the pathogenesis of glomerular disease and be targets for therapeutic interventions. The purpose of the present review is to summarize current knowledge of membrane transporters in the podocyte and to give examples of transporter activity in health and disease.

## 2. Epithelial Transporters in the Glomerulus

As described in the introduction, renal membrane transporters have been extensively studied in the proximal tubule, largely in relationship to drug metabolism and secretion. The classification of these transporters has traditionally been divided according to the electrical charge of the solute at physiologic pH: organic anion transporters (OATs), organic cation transporters, (OCTs), zwitterion transporters (OCTNs), and neutral compound transporters. Several of the charged solute transporters belong to the solute carrier protein super-family 22 (SLC22); mutations in the SLC22 genes that encode the transporters can lead to specific diseases such as renal hypouricemia and are associated with Crohn’s Disease and gout [5,6]. Also, drug-drug interactions at the transporter can alter pharmacokinetics and lead to drug toxicity. A second tubular transport family is the ATP-binding cassette (ABC) family, which includes the important efflux transporter P-glycoprotein (PGP). The same family is also charged with lipid transport including cholesterol efflux; mutations in a substantial number of the 48 human ABC transporters have been linked to human disease [7]. Other transporter families include SLC21, 44 and 47. The products of the SLC21 family, known as organic anion-transporting polypeptides (OATPs) also transport cations, zwitterions and neutral substances [8,9]. It has been shown that uptake and efflux are coexpressed in tissues important for drug metabolism; a number of OATP substrates are shared with efflux transporters, and the coordinate activity between the uptake transporter and the efflux transporter determine the net cellular drug entry or efflux of shared substrates [8]. In polarized cells such as those of the proximal tubular epithelium or the liver, the uptake transporter is found on the basolateral surface of the plasma membrane, whereas the efflux transporter is found on the apical surface to deliver the substrate to the urinary space in the kidney or to the bile in the liver.

Probably the best studied transporter classification is that of OATs; hippurate is the dominant aromatic anion excreted in the urine, and para-aminohippurate (PAH) has been used as a probe to study OAT kinetics for at least 75 years [4,10]. PAH secretion has been used to study renal blood flow and the anatomic sites of transport. A relevant finding of these studies is that nephron PAH transport is not heterogeneous along the course of the tubule and transporters are not distributed randomly along the course of the tubule [4,11]. Other endogenous OAT substrates in the proximal tubule include bile salts, cyclic AMP, prostaglandins, and other metabolic substances. In the setting of renal failure there is an enhancement of organic acid secretion as aromatic acids accumulate, and the secretory capacity of each surviving nephron is increased as an adaptive response [12]. Some examples of exogenous OAT substrates include antibiotics (particularly beta-lactam antibiotics such as penicillins and cephalosporins), loop diuretics (in fact the secretion of furosemide into the tubular lumen is necessary for the drug to effect its action), and radiocontrast media. OCTs have been less studied in the proximal tubule although this is hardly a reflection of their relative importance. A vast number of endogenous cationic substances and drugs are secreted by the proximal tubule [4]: creatinine, dopamine, histamine, morphine, atropine, tetraethylammonium, *etc.* Several large well-written recent reviews of OATs and OCTs exist in the literature [13,14,15,16], and further discussion of interesting proximal tubule physiology is outside of the scope of our glomerular review. Nevertheless, the juxtaposition of the glomerulus and the proximal tubule and the similar origin from the metanephric mesenchyme [17] suggest that proximal tubule and podocyte transport mechanisms are likely to be shared.

Podocyte morphology is complex. The cell body sends out primary branches which in turn produce fine secondary branches, known as foot processes, which wrap around the glomerular capillary and interdigitate with foot processes of neighboring podocytes [18,19,20]. A very fine membrane forms between adjacent foot processes—the slit diaphragm. The principal structural component of the slit diaphragm is a large molecular weight zipper-like protein known as nephrin [21,22]. Genetically-determined absence of nephrin, as occurs in congenital nephrotic syndrome of the Finnish type, leads to severe and lethal protein losses in the urine. Nephrin also acts as a scaffold for other important podocyte proteins which are necessary for the protein barrier function of the podocyte, such as podocin, zonula occludens protein (ZO-1), CD2-associated protein [23], *etc.* Through its connection with synaptopodin, nephrin also acts as an anchor for actin filaments which subserve the contractile function of the podocyte [24]. The podocyte produces a number of cytokines and autocrine and paracrine hormones, such as IL-6, IL-8 and vascular endothelial growth factor (VEGF) [25]. The foot processes are anchored to the basement membrane by a number of adherence molecules and podocyte cell bodies are attached by epithelial tight junctions. Thus, for plasma ultrafiltrate to reach the glomerular urinary space, it must traverse the capillary endothelium, basement membrane and the slit diaphragm. The two principal proteinuric glomerulopathies which affect the podocyte—focal segmental glomerular sclerosis (FSGS) and minimal change disease (MCD)—are both characterized ultrastructurally in the early stages of disease by loss of the fine foot process anatomy and detachment from the underlying basement membrane [26]. Thus, the integrity of podocyte anatomy is essential for its role in the glomerular protein barrier. In fact, as in other highly specialized and morphologically complex cells, podocytes have limited mitotic capacity and repair mechanisms, and are thus particularly vulnerable to injury and to scarring. The maintenance of these highly differentiated cells requires coordinated uptake and efflux between the extracellular and intracellular spaces, mediated by small molecule transporters in health which may also be responsible for pharmacologic trafficking in these cells. 

To study podocyte pathology in the laboratory a number of models have been developed for whole animals, isolated glomeruli and cultured podocytes. Podocyte injury may be induced by injecting puromycin aminonucleoside (PA), adriamycin, lipopolysaccharide, protamine sulfate or other podocyte toxins in the whole animal or in the incubation medium of isolated glomeruli or cultured podocytes. Pippin *et al.* (2009) [27] have produced an elegant and practical guide to inducible rodent models of acquired podocyte diseases. By far the most common models involve PA and adriamycin. The precise mechanism of PA-induced injury remains unknown. Apparently, this small molecule causes direct DNA damage via the production of reactive oxygen species; rats pretreated with oxygen radical scavengers before receiving PA have less proteinuria and podocyte injury [28]. The progression of glomerular lesions from podocyte effacement, which would mimic the lesion of MCD, to glomerular scarring—FSGS—largely depends on cumulative PA dose and duration of administration. 

Although the importance of transporters in maintaining podocyte integrity is intuitively apparent, the number of related research papers is small (Table 1). In 2000, Gloy *et al.* [29] reported properties of neutral, acidic and basic amino acid transporters in cultured mouse podocytes. Using cell depolarization as a marker for cellular transport, they found that extracellular sodium was necessary for the uptake of most amino acids tested and that PA inhibited both depolarization and conductance induced by amino acids added to the podocyte bath. In the recent past Jung *et al.* [30] found an OAT isoform mOATLP1 in mouse kidney and liver by searching the expressed sequence tag database. The transporter mRNA renal tissue distribution was confined to the glomerular epithelial cells, the distal tubule and the collecting ducts; there was no staining in the proximal tubule or thick ascending limb of the nephron. Substrate specificity of this transporter could not be identified. Subsequently, using the same technique Lee *et al.* [31] found an organic cation like transporter (mOCTL1) in mouse parietal and visceral epithelial cells as well as the proximal, distal and collecting tubules. The transporter mRNA tissue distribution was confined to the kidney, but again substrate specificity could not be identified. In addition to these two studies microarray expression data obtained from mouse primary podocytes or mouse podocyte cell lines, publicly available as GEO data sets [32], show that podocytes express complete series of drug transporters of the OATP, ABC and SLC 22 families.

Two groups of researchers have found glomerular proteins which mediate reduced glutathione transport—important in situations of oxidative stress. In 2011 Quezada and colleagues [33] reported elevated mRNA expression of multidrug resistance-associated proteins (MRP/ABCC) 1, 3, 4 and 5 in rat glomeruli. Three weeks after induction of diabetes in streptozotocin-treated rats, a decline in reduced glutathione levels and an increase in the expression and activity of MRP1 (ABCC1) was observed. These lower glutathione levels were increased by *ex vivo* treatment with pharmacological inhibitors of MRP1 activity (MK571). The authors concluded that increased activity of MRP1 in diabetic glomeruli is correlated with an inadequate adaptive response to oxidative stress. Also Peng *et al.* [34] detected MRP in glomeruli of rodents and humans; the glomerular cell type was not specified in either paper.

The sodium-dependent inorganic phosphate transporters regulate phosphate influx from the extracellular milieu—a vital activity considering the necessity of phosphate to synthesize ATP. Sekiguchi *et al.* [35] studied the effect of the overexpression of the type III phosphate transporter Pit-1 on the glomerular permeability barrier. Pit-1 transgenic (TG) rats demonstrated increased proteinuria, hypoalbuminemia and dyslipidemia. Foot processes were effaced, and TG rat podocytes showed higher phosphate uptake than wild type rats, especially under low phosphate concentrations. Whereas constituent proteins such as nephrin, synaptopodin, and podocin were not different from control rats, injury markers such as desmin and connexin were markedly increased in TG rats. The authors concluded that overexpression of Pit-1 in rat glomeruli leads to podocyte injury and progression of glomerular sclerosis.

A very significant study in terms of clinical glomerular research was performed by Xia *et al.* [36], who identified the specific location and function of the OCT plasma membrane monoamine transporter (PMAT). PMAT belongs to the equilibrative nucleoside transporter (ENT) family (SLC29) and transports organic cations and the purine nucleoside adenosine by a sodium independent, pH sensitive mechanism. By developing a polyclonal antibody to a specific amino acid sequence of the PMAT protein the investigators were able to localize the molecule predominantly to the podocyte. They further demonstrated that PA is transported by PMAT into the cell, and by potentiating the presence of PMAT in the podocyte, they could increase cell sensitivity to PA and by inhibiting PMAT they could abolish PA toxicity in PMAT-expressing cells. This observation defines an extremely important step to understand how PA—and conceivably other pathogens—exerts its toxic effect on the podocyte.

**Table 1:** Membrane transporters described in the podocyte.

Membrane Transporter	*Species*	Reference
Amino acid transporters	*Mus muculus* (mouse)	Gloy *et al.*, 2000 [29]
mOATPLP1	*Mus musculus* (mouse)	Jung *et al.*, 2006 [30]
mOCTL1	*Mus musculus* (mouse)	Lee *et al.*, 2007 [31]
PMAT	*Rattus norvegicus* (rat)	Xia *et al.*, 2009 [36]
Pit-1	*Rattus norvegicus* (rat)	Sekiguchi *et al.*, 2011 [35]
MRPs	*Homo sapiens* (human)/*Rattus norvegicus* (rat)	Quezada *et al.*, 2011 [35]; Peng *et al.*, 1999 [34]
OATPs/Pgp-glycoprotein	*Homo sapiens* (human)/*Rattus norvegicus* (rat)	Zennaro *et al.*, 2013 [32]

## 3. Podocyte Transporters: PA and Penicillin 

Our interest in small molecule podocyte membrane transport has developed during our 20 years of glomerular albumin permeability studies in a number of animal models, particularly in isolated rat glomeruli and more recently in the zebrafish [37]. Small molecule transporters may provide a portal for pathogens or alterations—genetic or acquired—of the transporters themselves may lead to podocyte-associated glomerulopathies. In a recent multifaceted study [32] we studied properties of penicillin G, which is a substrate of several transporters in the SLC families, in the context of podocyte injury induced by PA. The results showed that PA increased albumin permeability in isolated glomeruli and increased proteinuria in whole animals, and these effects were inhibited by preincubation or pretreatment with penicillin G in a dose-dependent manner. If the glomeruli were exposed to PA before penicillin G, albumin permeability remained elevated, suggesting that penicillin pretreatment blocked transport of PA into the cell. Likewise, PA reduced adherence of glomeruli on a collagen IV substrate, and penicillin inhibited this effect. In cultured podocytes penicillin preserved cell viability and cytoskeleton organization in podocytes subsequently exposed to PA. In human podocytes we found mRNA expression of OATP-A,B,D, and E, whereas OATP-C (liver specific), OAT3 and the peptide transporter PEPT1 were absent and PEPT2 showed a low level of expression. The findings in rat podocytes were analogous. Significant variations in mRNA expression induced by PA were observed only for Oatp2, which was increased in rat podocytes after PA treatment, but lesser expression alterations were also noted for OATP-A in human lines and Oatp1 in rat podocytes. Fluorescent penicillin G uptake activity of OATPs and Oatps were blocked by cyclosporin or by rifampicin, both known inhibitors of the OATs. 

**Figure 2:**
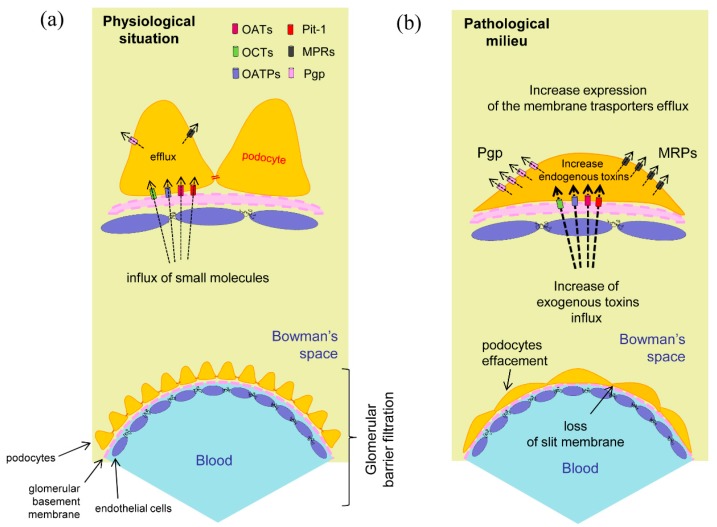
Podocytes membrane transporters. (**a**) Expression and hypothetical localization in physiological conditions. The membrane transporters regulate the influx and efflux of the specific compounds helping to maintain cellular integrity; (**b**) In the pathological situation the foot processes effacement may correlate to the increased input of toxic exogenous substances or to the appearance of cellular metabolites due to a previous cell damage. The presence of intracellular toxic compounds could promote the raise of efflux membrane transporters expression for the elimination of these substances [32,33].

Efflux activity of the transporter P-glycoprotein (PGP) in the podocyte (see below) was also studied intensely with immunofluorescence and flow cytometry analysis. The PGP efflux transporter gene MDR1 was expressed by human podocytes and rat gene homologues were expressed in rat podocytes. Gene expression was increased by PA incubation, and PA also caused remodelling of the podocyte cytoskeleton as shown by the reduction of synaptopodin and by almost complete disappearance of actin stress fibers. A specific fluorescent substrate of PGP, Rho 123, was used to measure the activity of the transporter. PA incubation increased Rho 123 efflux compared to control podocytes, whereas cyclosporin A blocked this effect. Also, cyclosporin A prevented the PGP-mediated egress of fluorescent penicillin. We conclude that both uptake and efflux transporters are present in the podocyte membrane. Penicillin G and PA share these transport systems as substrates, and transporters play a role in PA-induced podocyte injury. The physiological expression in podocytes of the membrane transporter is significantly altered in pathological models as described in Figure 2. 

## 4. Membrane Transporters and Cell Pathology—The PGP Example

Our study confirmed previous reports of PGP as a key egress transporter and a likely player in podocyte pathogenesis. Its importance in drug metabolism was demonstrated in the 1990s with the manufacture of knockout mice [38]. A member of the ATP-binding cassette family, PGP is the product of the multidrug resistance gene mdr1 or 2, so called because PGP rapidly extrudes chemotherapeutic agents from cancer cells, rendering the drugs ineffective. In fact Rho 123 is used to screen cancer cells for natural and synthetic compounds that could compete with chemotherapeutic agents for excretion via PGP. In humans two genes—mdr1 and 2—encode PGP, whereas in rodents there are three genes—mdr1 (mdr1b), mdr2 and mdr3 (mdr1a) [39]. The genes are also referred to as Abcb1a, 1b, and Abcb4. PGP transports diverse substrates, including peptides, steroids and a number of hydrophobic molecules including calcium channel blockers and immunosuppressive agents. In 1994 Bello-Reuss and Ernest described PGP activity in human glomerular mesangial cells [40]. The interesting characteristic of the mesangial cell is that there is no polarity, and thus one assumes that the function of PGP is to extrude natural substrates from the cell interior, which may be harmful if allowed to accumulate. As in our experiments in the podocyte, efflux of Rho 123 efflux was inhibited by cyclosporin and in this case adriamycin toxicity was enhanced when administered together with cyclosporin. Thus, as in the podocyte the simultaneous exposure to two PGP substrates can be deleterious to mesangial cells.

Cvetkovic *et al.* [41] demonstrated in an experimental expression system that there is a coupling between OATP and PGP, such that drugs that alter PGP activity also affect the function of OATP transporters, as we have seen with cyclosporin in cultured podocytes. They also found that the kinetics of the Oatp transporters for the antihistamine drug fexofenadine varied among themselves. In this case the affinity (Km) of Oatp2 was almost 5-fold greater than for Oatp1, but the maximal rate of transport was over 10-fold smaller. Recently, Tramonti *et al.* [39] have shown that mdr1 in whole kidney and PGP expression in membrane-enriched fractions are increased in uninephrectomized rats. Interestingly, the expression of mdr1a was markedly upregulated by uninephrectomy—nearly 10-fold—whereas the increase in mdr1b was only about 50 per cent. At the level of the podocyte we ponder the possibility that the response to a discrete pathogen or to a drug therapy may vary from individual to individual according to the endowment of transporter gene polymorphisms. In fact in an interesting review of proposed mechanisms of steroid resistance in childhood nephrotic syndrome Swierczewska and colleagues [42] describe several studies in which polymorphisms of the MDR1 gene were correlated with steroid resistance.

Finally and very importantly, PGP is a key determinant of tissue levels of several drugs, including dexamethasone, cyclosporin and digoxin. Nearly 20 years ago Schinkel *et al.* [43] showed that digoxin and cyclosporin concentrations increased dramatically in brain tissue in mdr1a knockout mice compared to wild type animals, dexamethasone less so. Morphine tissue levels were not affected by the absence of PGP. The authors opined that the modification of drug levels seen in patients receiving multiple drugs, such as the enhancing effect of calcium channel blockers on the cyclosporin level, might not only be due to competition for metabolizing enzymes at the level of the liver but also to PGP inhibition at the apical membrane. They also noted a marked variation of drug concentrations in knockout mice depending on the tissue type. The expression of uptake and efflux transporters is naturally greater in the tubular portions of the nephron than in the glomerulus, but GEO data sets indicate clear genetic expression of transporter DNA in the podocyte.

## 5. Podocyte Transporters as Pharmacological Mediators

As mentioned previously, several investigators have established the podocyte as the prime target of both genetic and acquired pathology that leads to important proteinuric glomerulopathies, specifically minimal change disease (MCD) and focal segmental glomerular sclerosis (FSGS). Unfortunately, the pathogenesis of these two diseases, often collectively termed “idiopathic nephrotic syndrome” is unknown. The early ultrastructural hallmark of both diseases is loss of the fine secondary processes of the podocyte and eventual detachment from the underlying basement membrane [26,44]. An inflammatory component or antibody or complement deposition are absent. The podocyte injury progresses to focal sclerosis and often to chronic renal insufficiency requiring renal replacement therapy. After renal transplantation idiopathic nephrotic syndrome recurs in approximately 30 per cent of patients [45], and this phenomenon has led several investigators in the field to speculate that a circulating factor may initiate the disease process. 

Although no inflammatory component can be detected as can be seen in many autoimmune glomerulopathies such as systemic lupus erythematosus or Goodpasture’s syndrome, immunosuppressive drugs such as corticosteroids and cyclosporin are nevertheless effective in reducing proteinuria. This has historically been interpreted as evidence that the idiopathic nephrotic syndrome is due to an immunologic perturbation, assuming that the drugs are inhibiting a pathological process in lymphocytes or other steroid-sensitive cells. Recently, an alternative explanation has been advanced that some immunosuppressors have a direct action on podocytes without the requirement of an immunological mediator [46]. Using conditionally immortalized human podocytes, Xing *et al.* [47] found that the synthetic glucocorticoid dexamethasone in therapeutic concentrations upregulated expression of nephrin and tubulin-alpha (a microtubule protein) and downregulated VEGF. By downregulating the cyclin kinase inhibitor p21, which normally acts as a molecular “brake” on podocyte proliferation and repair, podocyte survival was enhanced by dexamethasone without any effect on apoptosis. Podocyte IL-6 production was suppressed by dexamethasone. There was a faint indication that glucocorticoid receptors were increased in cytoplasmic and nuclear lysates in the presence of dexamethasone, which would be at odds with the known negative feedback mechanism associated with glucocorticoid treatment. Thus, the authors concluded that corticosteroids could produce a direct antiproteinuric effect on podocytes through multiple pathways without the requirement of engaging the immune system. Also operating on the assumption that steroids have a direct effect on the podocyte, Wada *et al.* [48] demonstrated that dexamethasone inhibited apoptosis in the presence of PA. The mechanisms involved in the prevention of apoptosis by dexamethasone included downregulation of the tumor suppressor protein p53 which normally activates gene transcription, inhibition of proapoptotic protein Bax, increasing expression of the antiapoptotic proteins Bcl-2 and Bcl-xL and inhibition of translocation of the apoptosis-inducing factor (AIF) from the cytoplasm to the nucleus.

In a sophisticated series of experiments Faul *et al.* [49] demonstrated that cyclosporine A, which classically inhibits cytokine production and T lymphocyte proliferation by binding to and inhibiting the cytosolic phosphatase calcineurin, thereby preventing the translocation of the nuclear factor of activated T cells (NFAT) to the nucleus, also has a direct protective effect on the podocyte. Calcineurin is present in all cell types, including podocytes, and one of its substrates is the actin-organizing protein synaptopodin. By inhibiting calcineurin, cyclosporin increased stress fiber content and synaptopodin expression and reduced proteinuria induced by lipopolysaccharide in mice. Finally, it has been known for some time that the thiazolidinediones (TZD) pioglitazone (Pio) and rosiglitazone (Rosi), widely used for the treatment of type 2 diabetes, reduce proteinuria and podocyte injury in both diabetic nephropathy and nondiabetic glomerulosclerosis in mouse and rat models and in humans. Whereas the uptake of lipophilic cyclosporine (and fellow calcineurin inhibitor tacrolimus) and glucocorticoids into the cell is thought to occur by passive diffusion or by endocytosis [50], the egress of the drugs, at least in liver and brain, is definitely dependent on PGP. 

Recently, Agrawal *et al.* [51] clarified the mechanism by incubating differentiated mouse podocytes with Pio, Rosi or dexamethasone with PA, either simultaneously or successively. All three drugs increased podocyte viability to near normal levels. PA treatment resulted in substantial loss of actin filaments in surviving cells; treatment with the TZDs and with dexamethasone preserved the actin filaments although the organization of the fibers was different from control cells, forming “subcortical ring-like structures”. Rosi also decreased various podocyte mitogen-activated protein kinases (MAPK); Pio did not. The TZDs increased glucocorticoid receptor transcription and phosphorylation but did not cause downregulation of the receptor. Interestingly, as opposed to cyclosporine in the previous study by Faul *et al.* [49], the TZDs and dexamethasone all significantly increased podocyte calcineurin activity. Obviously, there is still much work needed to clarify the protective effects of standard therapies—glucocorticoids and calcineurin inhibitors—at the level of the podocyte.

The back story of the action of the TZDs is fascinating. This class of drugs acts as a potent agonist to the nuclear receptor peroxisome proliferator-activated receptor-gamma (PPARγ), and has been used clinically as an insulin sensitizer in patients with type 2 diabetes mellitus. The PPARs are members of the nuclear receptor superfamily of ligand-inducible transcription factors. By binding to responsive regulatory elements in the nucleus the PPARs control the expression of networks of genes involved in adipogenesis, lipid metabolism, inflammation and maintenance of glucose homeostasis. The PPARs are ubiquitously expressed in body tissues, but PPARγ especially is known as “the master regulator” of adipocyte differentiation, as PPARγ null mice are completely devoid of adipose tissue. Ahmadian *et al.* have recently produced a very interesting review of the protean effects of PPARγ and its therapeutic implications for the future [52]. Oliver *et al.* [53] have developed a potent PPARδ agonist, GW501516. In macrophages, fibroblasts, and intestinal cells GW501516 increased expression of the reverse cholesterol transporter ATP-binding cassette A1 (ABCA1), inducing apolipoprotein A1-specific cholesterol efflux and increased high density lipoprotein concentrations. It is conceivable that other PPAR agonists have a similar action on uptake and efflux transporters. As a case in point hereditary metabolic diseases such as Tangier disease or familial hypoalphalipoproteinemia are associated with loss-of-function mutations in the ABCA1 gene. These patients have low levels of high density lipoproteins and elevated triglycerides and show an increased incidence of cardiovascular disease. The authors speculate that therapies that increase the expression of ABCA1 could provide a new approach to treating atherogenic dyslipidemia. Zuo and colleagues [54] found that Pio, given at the time or after but not before PA, reduced proteinuria, restored synaptopodin and tended to improve foot process effacement. There was no significant difference in glomerular filtration, effective circulating volume, blood pressure or fractional sodium excretion. PAN-injured podocytes had decreased PPARγ, less nephrin and α-actinin-4 and more apoptosis. Whereas the investigators measured sodium channel and aquaporin presence in the collecting ducts of PA-injured and Pio-treated animals (unchanged in both groups compared to controls), unfortunately we have no information about podocyte transporter status.

## 6. Conclusions

**Figure 3:**
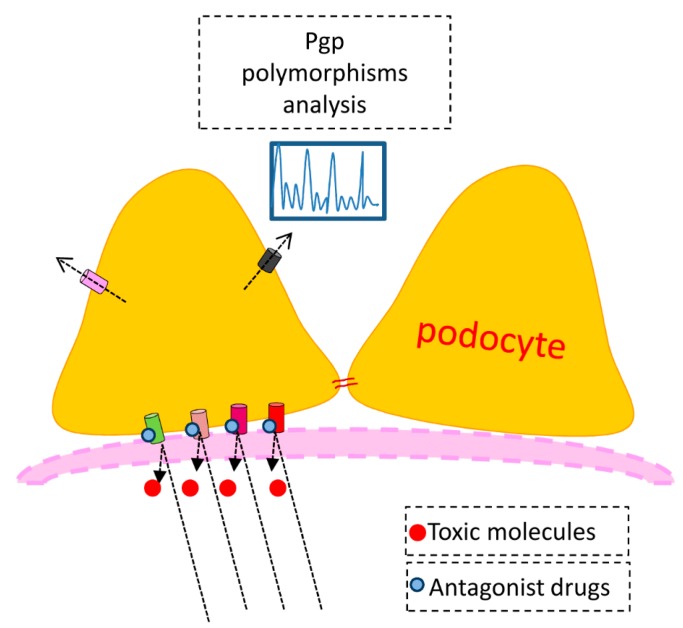
Proposed working model. One could hypothesize the use of specific antagonists to block the entry into the cell of exogenous toxic substances. It can be assumed that the response to a drug therapy may vary from individual to individual according to the endowment of Pgp polymorphisms. Influx transporters: in green OCTs, in pink OATPs, in violet OATs and in red Pit-1; Efflux transporters: in pink Pgp and in dark gray MPRs.

Considerable research effort has been directed toward understanding the barrier to macromolecular filtration provided by the component of the slit diaphragm, but little attention has been paid to the potential of podocytes to handle plasma proteins beyond the its specialized morphological features. We propose that membrane transporters in the podocyte are important in normal physiologic processing of metabolic events, that they are necessary for transport and efflux of therapeutic pharmaceuticals, and importantly may be mediators for entrance of pathogens into the podocyte or may themselves be targets of pathogenetic processes or genetic anomalies which lead to proteinuric diseases. There are likely to be numerous nephrotoxins which affect specific transport activity which should be studied. Further research is necessary at the molecular level to clarify the role of transporters in disease processes and some lines of research are proposed in the Figure 3.
